# Comparison of the Schwartz and CKD-EPI Equations for Estimating Glomerular Filtration Rate in Children, Adolescents, and Adults: A Retrospective Cross-Sectional Study

**DOI:** 10.1371/journal.pmed.1001979

**Published:** 2016-03-29

**Authors:** Luciano Selistre, Muriel Rabilloud, Pierre Cochat, Vandréa de Souza, Jean Iwaz, Sandrine Lemoine, Françoise Beyerle, Carlos E. Poli-de-Figueiredo, Laurence Dubourg

**Affiliations:** 1 Exploration Fonctionnelle Rénale et Métabolique, Groupement Hospitalier Est Hôpital Edouard Herriot, Hospices Civils de Lyon, Lyon, France; 2 Coordenação de Aperfeiçoamento do Pessoal de Nível Superior (CAPES), Brasilia, Brazil; 3 Programa de Pós-graduação em Medicina e Ciências da Saúde, Pontifícia Universidade Católica do Rio Grande do Sul, Porto Alegre, Brazil; 4 Programa de Pós-graduação em Ciências da Saúde, Universidade de Caxias do Sul, Caxias do Sul, Brazil; 5 Service de Biostatistique, Hospices Civils de Lyon, Lyon, France; 6 Université de Lyon, Lyon, France; 7 CNRS UMR 5558, Laboratoire de Biométrie et Biologie Evolutive, Equipe Biostatistique-Santé, Villeurbanne, France; 8 UMR 5305, Biologie Tissulaire et Ingénierie Thérapeutique, Université Claude Bernard, Lyon, France; 9 Centre de Référence des Maladies Rénales Rares, Service de Néphrologie et Rhumatologie Pédiatriques, Hospices Civils de Lyon, Lyon, France; 10 INSERM UMR 1060, Université Claude Bernard Lyon I, Lyon, France; 11 Laboratoire de Biochimie et Biologie Moléculaire, Groupement Hospitalier Est Hôpital Edouard Herriot, Hospices Civils de Lyon, Lyon, France; Instituto Mario Negri, ITALY

## Abstract

**Background:**

Estimating kidney glomerular filtration rate (GFR) is of utmost importance in many clinical conditions. However, very few studies have evaluated the performance of GFR estimating equations over all ages and degrees of kidney impairment. We evaluated the reliability of two major equations for GFR estimation, the CKD-EPI and Schwartz equations, with urinary clearance of inulin as gold standard.

**Methods and Findings:**

The study included 10,610 participants referred to the Renal and Metabolic Function Exploration Unit of Edouard Herriot Hospital (Lyon, France). GFR was measured by urinary inulin clearance (only first measurement kept for analysis) then estimated with isotope dilution mass spectrometry (IDMS)–traceable CKD-EPI and Schwartz equations. The participants’ ages ranged from 3 to 90 y, and the measured GFRs from 3 to 160 ml/min/1.73 m^2^. A linear mixed-effects model was used to model the bias (mean ratio of estimated GFR to measured GFR). Equation reliability was also assessed using precision (interquartile range [IQR] of the ratio) and accuracy (percentage of estimated GFRs within the 10% [P10] and 30% [P30] limits above and below the measured GFR). In the whole sample, the mean ratio with the CKD-EPI equation was significantly higher than that with the Schwartz equation (1.17 [95% CI 1.16; 1.18] versus 1.08 [95% CI 1.07; 1.09], *p <* 0.001, *t*-test). At GFR values of 60–89 ml/min/1.73 m^2^, the mean ratios with the Schwartz equation were closer to 1 than the mean ratios with the CKD-EPI equation whatever the age class (1.02 [95% CI 1.01; 1.03] versus 1.15 [95% CI 1.13; 1.16], *p <* 0.001, *t*-test). In young adults (18–40 y), the Schwartz equation had a better precision and was also more accurate than the CKD-EPI equation at GFR values under 60 ml/min/1.73 m^2^ (IQR: 0.32 [95% CI 0.28; 0.33] versus 0.40 [95% CI 0.36; 0.44]; P30: 81.4 [95% CI 78.1; 84.7] versus 63.8 [95% CI 59.7; 68.0]) and also at GFR values of 60–89 ml/min/1.73 m^2^. In all patients aged ≥65 y, the CKD-EPI equation performed better than the Schwartz equation (IQR: 0.33 [95% CI 0.31; 0.34] versus 0.40 [95% CI 0.38; 0.41]; P30: 77.6 [95% CI 75.7; 79.5] versus 67.5 [95% CI 65.4; 69.7], respectively). In children and adolescents (2–17 y), the Schwartz equation was superior to the CKD-EPI equation (IQR: 0.23 [95% CI 0.21; 0.24] versus 0.33 [95% CI 0.31; 0.34]; P30: 88.6 [95% CI 86.7; 90.4] versus 29.4 [95% CI 26.8; 32.0]). This study is limited by its retrospective design, single-center setting with few non-white patients, and small number of patients with severe chronic kidney disease.

**Conclusions:**

The results from this study suggest that the Schwartz equation may be more reliable than the CKD-EPI equation for estimating GFR in children and adolescents and in adults with mild to moderate kidney impairment up to age 40 y.

## Abstract 2

In this retrospective cross-sectional study, Luciano da Silva Selistre and colleagues compare Schwartz and CDK-EPI equations for estimating glomerular filtration rate in patients of different ages and degrees of renal impairment.

## Editors' Summary

**Background:**

Throughout life, our kidneys filter waste products (from food and from the normal breakdown of tissues) and excess water from our blood to make urine. If our kidneys stop working for any reason, the rate at which they filter the blood (the glomerular filtration rate, or GFR) decreases, and dangerous amounts of creatinine and other waste products build up in the blood. Kidneys can stop working suddenly, but in chronic kidney disease (CKD), a condition that affects more than 10% of the world’s population, kidney function declines gradually over many years. The symptoms of CKD, which rarely occur until the disease is very advanced, include tiredness, swollen feet, and frequent urination, especially at night. CKD cannot be cured, but its progression can be slowed by controlling high blood pressure and diabetes and by adopting a healthy lifestyle; the same interventions also reduce the chance of CKD developing in the first place.

**Why Was This Study Done?:**

CKD is linked with an increased risk of end-stage renal (kidney) disease and cardiovascular disease. Early identification of CKD can prevent these life-threatening complications, so clinical practice guidelines have been proposed for the diagnosis and management of CKD in the general population. The assessment of GFR is central to these guidelines. GFR can be measured by infusing inulin, a compound that is eliminated from the body by glomerular filtration, into the blood and measuring its rate of appearance in the urine. However, in routine clinical practice, GFR is usually estimated from blood creatinine levels using a GFR estimating equation (creatinine levels vary considerably within and between individuals, so an equation is needed to convert measured creatinine levels into GFR estimates). Examples of creatinine-based GFR estimation equations include the Chronic Kidney Disease Epidemiology Collaboration (CKD-EPI) equation and the Schwartz equation, which were developed in middle-aged adults and children, respectively. Few studies have evaluated the performance of such equations over all ages and levels of kidney impairment, so here the researchers assess the reliability of the CKD-EPI and Schwartz equations for estimating GFR in children, adolescents, and adults.

**What Did the Researchers Do and Find?:**

In 10,610 individuals referred to a single French hospital because of suspected or established kidney disease or before kidney donation, the researchers compared GFR measured by inulin clearance with GFR estimated using the CKD-EPI and Schwartz equations. They evaluated the reliability of each equation by calculating the average (mean) ratio of estimated GFR to measured GFR (a ratio of 1 indicates that the equation yielded GFR values identical to those from the gold-standard inulin clearance test) and by assessing the precision and accuracy of the estimated GFR values: the precision of a measurement indicates its reproducibility and reliability; the accuracy of a measurement indicates its closeness to the true value of a quantity. Across all the participants, the mean ratio of estimated GFR to measured GFR with the Schwartz equation was nearer to 1 than the mean ratio for the CKD-EPI equation. Among participants of all ages with measured GFR values indicating mild loss of kidney function, the mean ratio obtained with Schwartz equation was also nearer to 1 than that obtained with the CKD-EPI equation. Among young adults (18–40 years old) with measured GFR values indicating mild to moderate loss of kidney function, the Schwartz equation had better precision and was more accurate than the CKD-EPI equation, but, in all patients aged ≥65 years, the CKD-EPI equation performed better than the Schwartz equation. Finally, in children and adolescents, the Schwartz equation performed better than the CKD-EPI equation.

**What Do These Findings Mean?:**

Several aspects of this study (for example, its single-site setting and the low numbers of participants with severe CKD) may limit the accuracy and generalizability of its findings. However, these findings suggest that the Schwartz equation may be more reliable than the CKD-EPI equation for estimating GFR in children and adolescents and in adults up to 40 years old with mild to moderate kidney impairment. Up to now, there has been no consensus about when physicians should switch from using the Schwartz equation (which was developed in children) to using the CKD-EPI equation (which was developed in middle-aged adults) to estimate GFR in their patients. The findings of this study might therefore help physicians decide when to make this switch, thereby improving clinical decision-making and possibly helping to reduce the global burden of CKD.

**Additional Information:**

This list of resources contains links that can be accessed when viewing the PDF on a device or via the online version of the article at http://dx.doi.org/10.1371/journal.pmed.1001979.

- The UK National Health Service Choices website provides information for patients on chronic kidney disease, including information on screening for and diagnosing CKD and some personal stories about the disease
- The US National Kidney Foundation, a not-for-profit organization, provides information about chronic kidney disease and about the estimation of glomerular filtration rates (in English and Spanish)
- The US National Institute of Diabetes and Digestive and Kidney Diseases provides links to information about all aspects of kidney disease and information on creatinine-based GFR estimation equations; the US National Kidney Disease Education Program provides resources to help improve the understanding, detection, and management of kidney disease (in English and Spanish)
- World Kidney Day, a joint initiative of the International Society of Nephrology and the International Federation of Kidney Foundations, aims to raise awareness about kidneys and kidney disease
- Clinical guidelines for the evaluation and management of CKD produced by the Kidney Disease Improving Global Outcomes not-for-profit foundation are available
- MedlinePlus provides links to additional resources about kidney diseases

## Introduction

In the past decade, kidney disease has been recognized as a major public health burden. The prevalence of chronic kidney disease (CKD) currently exceeds 10% of the general population [1,2]. The two major hallmarks of CKD—reduced glomerular filtration rate (GFR) and increased urinary albumin excretion—are strong and graded prognostic factors of morbidity and mortality regardless of patient age, sex, ethnicity, or comorbidities. As preventing or slowing the progression of CKD toward end-stage renal disease relies mainly on early detection, international recommendations have been proposed for the diagnosis and management of CKD in the general population [1,3,4].

The assessment of GFR is currently the accepted surrogate marker of nephron endowment and a surveillance tool to monitor the progression of renal disease. Ideally, GFR should be measured by renal clearance of an exogenous marker that is exclusively eliminated by glomerular filtration (inulin, iohexol, chromium-51 ethylenediaminetetraacetic acid, etc.). However, for practical reasons, GFR measurement cannot be performed in routine clinical practice. Instead, serum levels of endogenous filtration markers, such as plasma creatinine (PCr), have traditionally been used to estimate GFR, along with urinary measurements in some cases [2]. Effective renal plasma flow is also an important tool for monitoring renal function, especially during the management of CKD or glomerular hyperfiltration [2–4]. However, effective renal plasma flow was not widely available at the time of the present study.

Thus, despite some drawbacks, isotope dilution mass spectrometry (IDMS)–calibrated PCr determination is now widely used as a noninvasive method for GFR measurement [2,4–6]. Indeed, though PCr determination is easy to perform, its results vary considerably between laboratories. To reduce this variation, all major manufacturers offer IDMS-calibrated PCr measurement procedures. Moreover, PCr concentration varies considerably within and between individuals because it depends on age, sex, muscle mass, nutritional state, diet, etc. [1,2,4]. This led to the development of several PCr-based GFR estimating equations in children and adults. These equations help physicians estimate GFR according to the patient’s characteristics and limit the uncertainty of an isolated PCr determination.

Among several GFR estimating equations, the Chronic Kidney Disease Epidemiology Collaboration (CKD-EPI) equation has been recommended in adults, and the Schwartz equation in children [3,4,7,8] (Table 1). Various studies have investigated the reliability of these equations at different CKD severity levels, but they did not investigate their reliability at different ages, especially at the transition from childhood to adulthood [9–13]. Actually, most adult PCr-based equations were developed in middle-aged or older individuals with various pathological conditions, not in young adults or adolescents [11,12,14,15]. Moreover, pediatric equations, such as the adjusted Schwartz equation, were developed in a small number of adolescents and only in individuals with mild to severe CKD [7].

**Table 1:** Formulas used for the estimation of glomerular filtration rate (ml/min/1.73 m^2^).

Equation	Condition	Formula
**CKD-EPI**	Female; PCr ≤ 61.88 μmol/l	eGFR = 144 × [PCr (μmol/l)/61.88]^−0.329^ × [0.993]^Age^ × [1.159 if black]
	Female; PCr > 61.88 μmol/l	eGFR = 144 × [PCr (μmol/l)/61.88]^−1.209^ × [0.993]^Age^ × [1.159 if black]
	Male; PCr ≤ 79.56 μmol/l	eGFR = 141 × [PCr (μmol/l)/79.56]^−0.411^ × [0.993]^Age^ × [1.159 if black]
	Male; PCr > 79.56 μmol/l	eGFR = 141 × [PCr (μmol/l)/79.56]^−1.209^ × [0.993]^Age^ × [1.159 if black]
**Schwartz**		eGFR = 36.5 × height (cm)/PCr (μmol/l)

To convert PCr values in μmol/l to mg/dl, divide by 88.4.

eGFR, estimated GFR.

In clinical practice, equation use in middle-aged individuals is a daily issue, and priority should be given to easy-to-use equations. Thus, there is a need for a comprehensive examination of the reliability of the most recommended PCr-based equations according to age [9,10]. The objectives of our study were the following: (1) to assess the reliability of the two most commonly used IDMS-calibrated PCr-based equations (i.e., Schwartz and CKD-EPI) and (2) to assess and compare the performance of these two equations across various age and GFR ranges.

## Methods

### Study Population

The study considered a cross-sectional retrospective sample of 10,610 individuals. It included all eligible individuals 3 to 90 y old referred between 22 July 2003 and 7 July 2014 to a single university department (namely, the Renal and Metabolic Function Exploration Unit of Edouard Herriot Hospital, Lyon, France) to undergo GFR measurement for suspected or established renal dysfunction or renal risk, or before kidney donation.

The exclusion criteria were as follows: (1) treatment by dialysis at the time of the study; (2) taking cimetidine, trimethoprim, or intravenous injections of albumin or diuretics before GFR measurement; (3) GFR >160 ml/min/1.73 m^2^; (4) GFR measurement by iohexol clearance.

The data were extracted from a database that included urinary clearance of inulin as measurement of GFR. In addition, the data included the clinical indication for the test, demographic data from a standardized interview, and measurements of blood pressure, pulse, and body mass index (BMI). Other blood and urine laboratory results were available in the hospital laboratory database.

**Fig 1:**
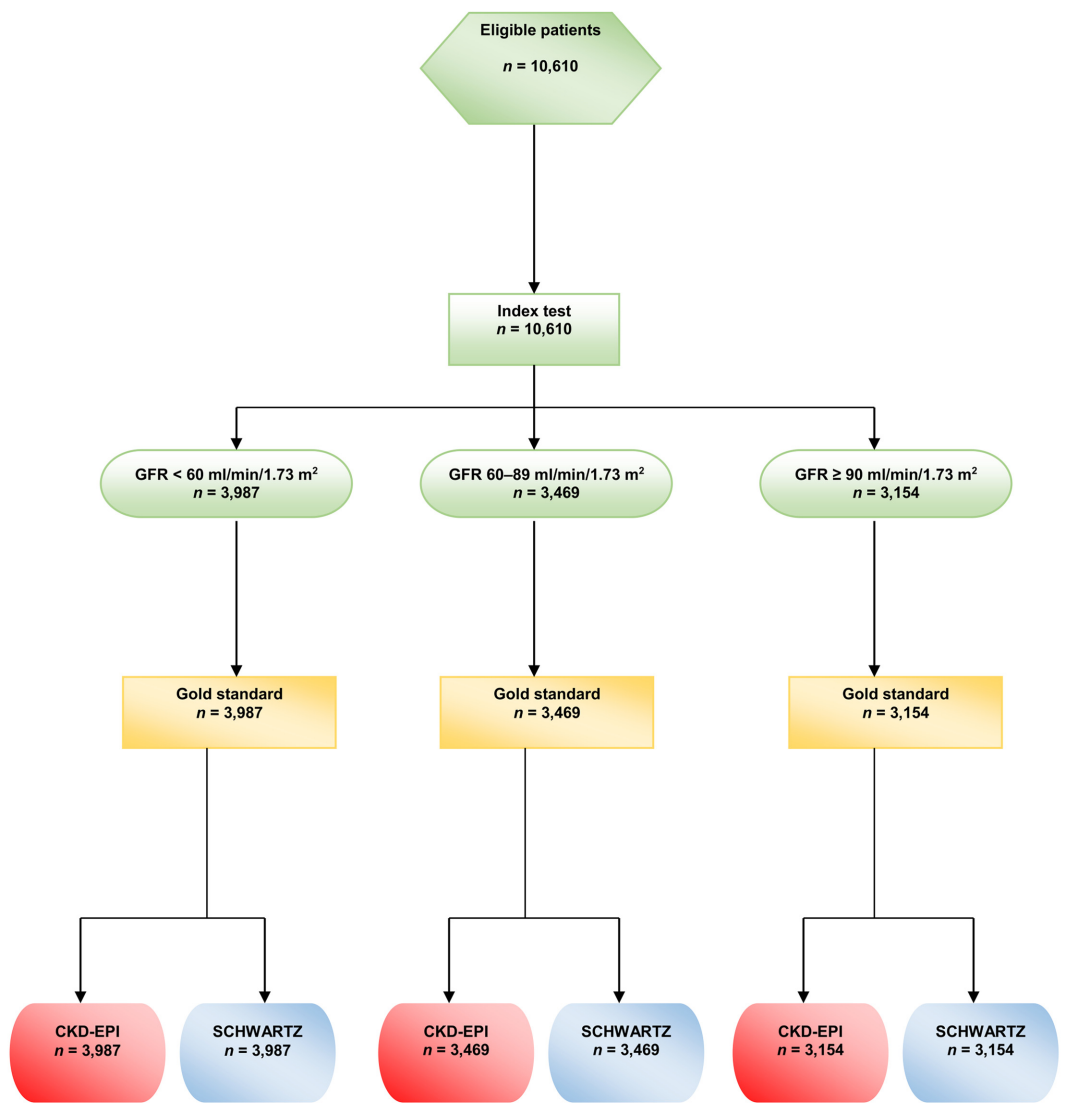
Flow chart of the study.

During the study period, some participants had several GFR measurements, but, for the present study, only the first GFR measurement in each participant was kept for analysis. (See the flow chart in Fig 1)

All the procedures were carried out in accordance with the ethical standards of institutional and/or national research committees and of the 1964 Helsinki Declaration and its later amendments or comparable ethical standards. Precisely, an appropriate written informed consent was obtained from all the participants or their legal representatives. The consent form contained information on the procedure itself as well as on the possibility of later use of the data for research purposes. According to current French law, an observational study that does not change the routine management of patients does not need to be declared or submitted to the opinion of a research ethics board ([16] and its subsequent amendments).

### Data Collection

Reliability assessment and comparisons between the two GFR equations were carried out on different age classes and different categories of measured GFR (mGFR). Based on previous studies on kidney physiology [17,18], the study considered five age classes: 2–12, 13–17, 18–40, 41–64, and ≥65 y. Various studies have shown that GFR declines steadily with age, starting at age 30–40 y, with an apparent acceleration after age 65–70 y [19]. Because our population included very different numbers of participants per age class in terms of Kidney Disease Improving Global Outcomes (KDIGO) categories IIIa, IIIb, and IV, we considered only three categories of renal function: <60, 60–89, and ≥90 ml/min/1.73 m^2^ [2].

Age, height, and weight were recorded. In participants <18 y old, the BMI was expressed as *z*-score according to the height-for-age and height-for-sex growth charts in France [20,21].

### Laboratory Assessments

Measurement of inulin clearance is still considered the gold standard for GFR measurement. Renal clearance of inulin was measured using a polyfructosan-based method (Inutest, Fresenius Kabi). A standard technique was used by a trained staff with a continuous infusion after a 30-mg/kg priming dose of polyfructosan. Water diuresis was induced by an initial oral administration of 5 ml/kg of water followed by 3 ml/kg every 30 min combined with an intravenous infusion of 0.9% sodium chloride. Three to four urine samples were collected, and a blood sample was drawn midway through each collection period. The clearance value, calculated by the usual *UV*/*P* formula, was the mean value of three to four clearance periods. The measurements of plasma and urine polyfructosan were performed using the same enzymatic method, which demonstrated very good specificity and reproducibility (within-run precision <1% and between-run precision <3.5%) [11]. The results were expressed per 1.73 m^2^ according to the Dubois formula: body surface area = height^0.725^ × weight^0.425^ × 0.007184.

All PCr measurements were performed with methods traceable to the National Institute of Standards and Technology creatinine standard reference (IDMS calibrated). From 10 October 2003 to 23 June 2010, PCr was measured by a kinetic, colorimetric, compensated Jaffé technique (Roche Modular); results were standardized by linear regression adjustment versus the concentrations obtained by liquid chromatography mass spectrometry. The calibration equation was as follows: standardized PCr = 0.9395 × Jaffé compensated serum creatinine (in μmol/l) + 4.6964. The coefficient of correlation was 0.97. From 24 June 2010, all PCr values were obtained by an enzymatic method traceable to the National Institute of Standards and Technology. According to KDIGO, the two techniques are considered similar [2]. PCr is expressed in μmol/l.

GFR estimation was carried out the same day inulin clearance was measured; estimated GFR (eGFR) was expressed in ml/min/1.73 m^2^ and was calculated with two PCr-based equations: CKD-EPI and Schwartz (Table 1) [2].

Albuminuria was expressed as the ratio of urine albumin to urine creatinine, and the participants were separated into three categories: (i) normal: <3 mg/mmol (<30 mg/g), (ii) increased: 3–30 mg/mmol (30–300 mg/g), and (iii) high: >30 mg/mmol (300 mg/g).

### Statistical Analyses

The bias, precision, and accuracy of the Schwartz and CKD-EPI equations were estimated for each age class and each category of renal function. Bias was defined as the mean of the eGFR/mGFR ratio. In a first step, the eGFR/mGFR ratio was modeled according to a linear mixed-effects model with random intercept to quantify the effect of the equation type (CKD-EPI or Schwartz equation) on the bias. The mean ratios according to the two equations were compared by a *t*-test in the linear mixed-effects model. In a second step, two models were built: a first model that included the variables “equation type” and “age class” and a second model that included an interaction between the variables “equation type” and “age class.” The second model allows quantification of the change of the effect of the equation type according to age. An ANOVA was used to compare the two nested models and draw conclusions regarding the statistical significance of the interaction. This analysis was carried out on the entire sample and on each category of renal function.

Precision was defined as the interquartile range (IQR) of the eGFR/mGFR ratio. Following the KDIGO guidelines, accuracy was defined at two levels: P10, the percentage of eGFR values within the 10% percent limits above and below the mGFR, and P30, the percentage of eGFR values within the 30% percent limits above and below the mGFR [2]. The 95% CIs of the IQR and accuracy values were obtained by bootstrap using the percentiles method. This method consists in taking the 2.5th and the 97.5th percentiles of the distribution of the IQR and accuracy values estimated on 2,000 bootstrap samples [22]. We used the ratio of eGFR to mGFR, instead of the difference, to assess the bias and precision of the two equations because the between-individual heterogeneity of the difference increased with GFR value. The use of the ratio allowed us to obtain a constant heterogeneity.

The analyses were performed with R for Windows, version 3.1.1 (http://cran.r-project.org/). The nominal *p*-value used to determine statistical significance was *p* < 0.05. (For STARD checklist and the full statistical plan see S1 and S2 Texts).

## Results

### Participants’ Characteristics

The clinical characteristics of the 10,610 participants are shown in Table 2. The median age of the participants was 50.8 y. Within the age range 3–90 y, 1,180 participants (11.0%) were less than 18 y, and 2,326 (22.0%) were aged 18 to 40 y. Among adult participants (≥18 y), 606 (6.4%) had a BMI < 18.5 kg/m^2^, and 441 (4.7%) had a BMI ≥35 kg/ m^2^.

**Table 2:** Characteristics of the patients in the different mGFR categories.

Characteristic	Whole Sample	mGFR Category		
		<60 ml/min/1.73 m^2^	60–89 ml/min/1.73 m^2^	≥90 ml/min/1.73 m^2^
**Number of participants**	10,610 (100.0%)	3,987 (37.6%)	3,469 (32.7%)	3,154 (29.7%)
**Age of adults, years**	53.4 [41.2; 62.7]	58.3 [47.8; 67.7]	51.9 [38.3; 60.7]	42.9 [31.9; 52.2]
**Age of children and adolescents, years**	11.0 [7.4; 14.8]	13.1 [8.6; 15.2]	11.4 [8.2; 15.1]	10.5 [7.1; 14.6]
**Age class**				
2–12 y	750 (7.0%)	58 (1.4%)	151 (4.4%)	541 (17.1%)
13–17 y	430 (4.0%)	61 (1.5%)	113 (3.2%)	256 (8.1%)
18–40 y	2,326 (22.0%)	523 (13.1%)	731 (21.1%)	1,072 (34.0%)
41–64 y	5,246 (49.5%)	2,067 (52.0%)	1,966 (56.7%)	1,213 (38.5%)
≥65 y	1,858 (17.5%)	1,278 (32.0%)	508 (14.6%)	72 (2.3%)
**Females**	4,944 (46.6%)	1,753 (44.0%)	1,681 (48.4%)	1,510 (47.8%)
**Kidney transplanted**	1,550 (14.6%)	958 (24.0%)	508 (14.6%)	84 (2.7%)
**Candidates for kidney donation**	193 (1.8%)	7 (0.2%)	67 (1.9%)	119 (3.8%)
**Weight of adults, kg**	68.0 [57.5; 79.50]	69.0 [59.0; 80.5]	66.0 [56.0; 78.0]	67.0 [57.0; 79.0]
Females	60.0 [52.0; 70.0]	61.0 [53.0; 72.0]	59.0 [51.0; 68]	59.0 [52.0; 69.0]
Males	74.0 [65.0; 85.0]	74.0 [65.0; 85.0]	74.0 [64.5; 84.0]	74.0 [65.0; 85.0]
**Height of adults, cm**	166.0 [159.0; 173.0]	166.0 [158.0; 173.0]	166.0 [159.0; 173.0]	167.0 [160.5; 174.0]
Females	160.0 [155.0; 164.5]	160.0 [155.5; 165.0]	160.0 [155.5; 165.0]	161.0 [157.0; 165.5]
Males	172.0 [167.0; 177.0]	171.0 [166.0; 176.0]	172.0 [167.0; 177.0]	173.0 [168.0; 178.0]
**BMI *z*-score of children and adolescents**	−0.2414 [−0.7457; 0.5601]	−0.3328 [−0.7197; 0.6329]	−0.2883 [−0.7595; 0.5400]	−0.2155 [−0.7456; 0.5396]
**Body surface area, m** ^**2**^	1.72 [1.55; 1.90]	1.77 [1.62; 1.93]	1.74 [1.59; 1.90]	1.76 [1.61; 1.93]
**BMI of adults, *n***	9,430	3,868	3,205	2,357
Median [IQR]	24.3 [21.3; 27.9]	25.0 [21.9; 28.7]	23.9 [21.1; 27.2]	23.8 [21.0; 27.2]
<18.5 kg/m^2^	606 (6.4%)	228 (5.8%)	203 (6.3%)	175 (7.5%)
≥35.0 kg/m^2^	441 (4.7%)	251 (6.4%)	106 (3.3%)	84 (3.6%)
**PCr, μmol/l** *	87 [66; 118]	128 [103; 142]	82 [69; 98]	62 [51; 73]
**mGFR, ml/min/1.73 m** ^**2**^	70 [48; 94]	42 [31; 52]	74 [66; 81]	106 [97; 117]
**Albuminuria** ** **, mg/mmol**	2.21 [0.76; 12.81]	6.92 [1.69; 42.12]	1.47 [0.63; 6.16]	1.19 [0.61; 3.75]
**Albuminuria** ** **category**				
<3 mg/mmol (<30 mg/g)	5,861 (55.2%)	1,380 (34.6%)	2,225 (64.1%)	2,255 (71.5%)
3–30 mg/mmol (30–300 mg/g)	2,957 (27.9%)	1,448 (36.3%)	856 (24.7%)	653 (20.7%)
>30 mg/mmol (300 mg/g)	1,793 (16.9%)	1,159 (29.1%)	388 (11.2%)	246 (7.8%)

Data are given as *n* (percent) or median [IQR]. Adults are individuals aged ≥18 y; children and adolescents are individuals aged 2–17 y.

*To convert PCr values to mg/dl, divide by 88.4.

**Ratio of urine albumin to urine creatinine.

The population consisted of three major groups of indication for GFR measurement: 193 candidates for living kidney donation, 8,867 cases of native CKD, and 1,550 kidney transplant recipients. The mean ± standard deviation of mGFR was 72.0 ± 32.0 ml/min/1.73 m^2^. Within the mGFR range of 3–160 ml/min/1.73 m^2^, 37.6% of the participants had values <60, 32.7% had values from 60 to 89, and 29.7% had values ≥90 ml/min/1.73 m^2^. The participants with mGFR values < 60 ml/min/1.73 m^2^ were older and had higher albuminuria values than the others. In the whole sample 1,793 participants (16.9%) had a ratio of urine albumin to urine creatinine > 30 mg/mmol (300 mg/g).

### Modeling of Bias According to Equation Type and Age

In the whole sample, the mean eGFR/mGFR ratio according to the CKD-EPI equation was significantly higher than the mean eGFR/mGFR ratio according to the Schwartz equation (1.17 [95% CI 1.16; 1.18] versus 1.08 [95% CI 1.07; 1.09], *p <* 0.001, *t*-test). The interaction between the type of equation and age class was statistically significant (*p <* 0.001, ANOVA). This indicates that the mean ratios given by the two equations have different changes with increasing age class (Table 3). With the Schwartz equation, the mean ratio was close to 1 in children and adolescents (2–17 y) and young adults (18–40 y) (1.03 [95% CI 1.01; 1.04] and. 0.96 [0.90; 1.01], respectively) but high in participants aged 65 y and older (1.24 [95% CI 1.18; 1.30]). With the CKD-EPI equation, the mean ratio was high in children but decreased with increasing age; it was closer to 1 than the mean ratio from the Schwartz equation in participants older than 65 y (0.93 [95% CI 0.89; 0.97]) (Table 3).

**Table 3:** Estimation of bias (mean eGFR/mGFR ratio) with the CKD-EPI and Schwartz equations according to age class in the whole sample and in different mGFR categories.

mGFR Category	Age Class	eGFR/mGFR Ratio (95% CI)	
		CKD-EPI Equation	Schwartz Equation
**Whole sample**	All participants	1.17 (1.16; 1.18)	1.08 (1.07; 1.10)
	<18 y	1.54 (1.51; 1.56)	1.03 (1.01; 1.04)
	02–12 y	1.64 (1.61; 1.66)	1.04 (1.01; 1.06)
	13–17 y	1.40 (1.35; 1.44)	1.01 (0.94; 1.08)
	All adults	1.13 (1.12; 1.14)	1.08 (1.07; 1.10)
	18–40 y	1.14 (1.12; 1.16)	0.96 (0.90; 1.01)
	41–64 y	1.07 (1.03; 1.11)	1.09 (1.06; 1.14)
	≥65 y	0.93 (0.89; 0.97)	1.24 (1.18; 1.30)
**<60 ml/min/1.73 m** ^**2**^	All participants	1.26 (1.24; 1.28)	1.26 (1.24; 1.28)
	<18 y	2.52 (2.34; 2.65)	1.27 (1.15; 1.40)
	02–12 y	2.51 (2.38; 2.66)	1.28 (1.16; 1.40)
	13–17 y	1.69 (1.57; 1.80)	1.24 (0.96; 1.53)
	All adults	1.29 (1.25; 1.33)	1.13 (1.09; 1.17)
	18–40 y	1.45 (1.27; 1.62)	1.13 (0.87; 1.38)
	41–64 y	1.27 (1.11; 1.45)	1.25 (1.00; 1.50)
	≥65 y	1.14 (0.97; 1.31)	1.34 (1.10; 1.59)
**60–89 ml/min/1.73 m** ^**2**^	All participants	1.15 (1.13; 1.16)	1.02 (1.01; 1.03)
	<18 y	1.76 (1.73; 1.79)	1.10 (1.06; 1.13)
	02–12 y	1.92 (1.88; 1.96)	1.11 (1.07; 1.15)
	13–17 y	1.57 (1.50; 1.63)	1.08 (0.98; 1.18)
	All adults	1.10 (1.09; 1.11)	1.01 (1.00; 1.03)
	18–40 y	1.32 (1.25; 1.39)	0.96 (0.87; 1.04)
	41–64 y	1.17 (1.10; 1.24)	1.03 (0.95; 1.11)
	≥65 y	1.08 (1.01; 1.15)	1.03 (0.94; 1.12)
**≥90 ml/min/1.73 m** ^**2**^	All participants	1.09 (1.08; 1.10)	0.92 (0.91; 0.93)
	<18 y	1.43 (1.42; 1.45)	1.02 (1.01; 1.04)
	02–12 y	1.46 (1.44; 1.48)	0.99 (0.97; 1.01)
	13–17 y	1.19 (1.17; 1.21)	0.92 (0.90; 0.94)
	All adults	1.00 (0.99; 1.02)	0.91 (0.90; 0.95)
	18–40 y	1.07 (1.06; 1.09)	0.87 (0.86; 0.89)
	41–64 y	1.00 (0.99; 1.04)	0.86 (0.85; 0.88)
	≥65 y	0.88 (0.87; 0.90)	0.97 (0.95; 0.98)

Results of the linear mixed-effects modeling.

The interaction between the equation type and the age class was statistically significant in the three categories of mGFR. Whatever the mGFR category, the mean eGFR/mGFR ratio with the CKD-EPI equation was high in children and adolescents (<18 y) (1.54 [95% CI 1.51; 1.56]). In the mGFR category 60–89 ml/min/1.73 m^2^, the mean eGFR/mGFR ratios with the Schwartz equation were closer to 1 than the mean eGFR/mGFR ratios with the CKD-EPI equation for participants of all ages (1.02 [95% CI 1.01; 1.03] versus 1.15 [95% CI 1.13; 1.16], respectively, *p <* 0.001, *t*-test) (Table 3).

### Equation Precision and Accuracy

**Fig 2:**
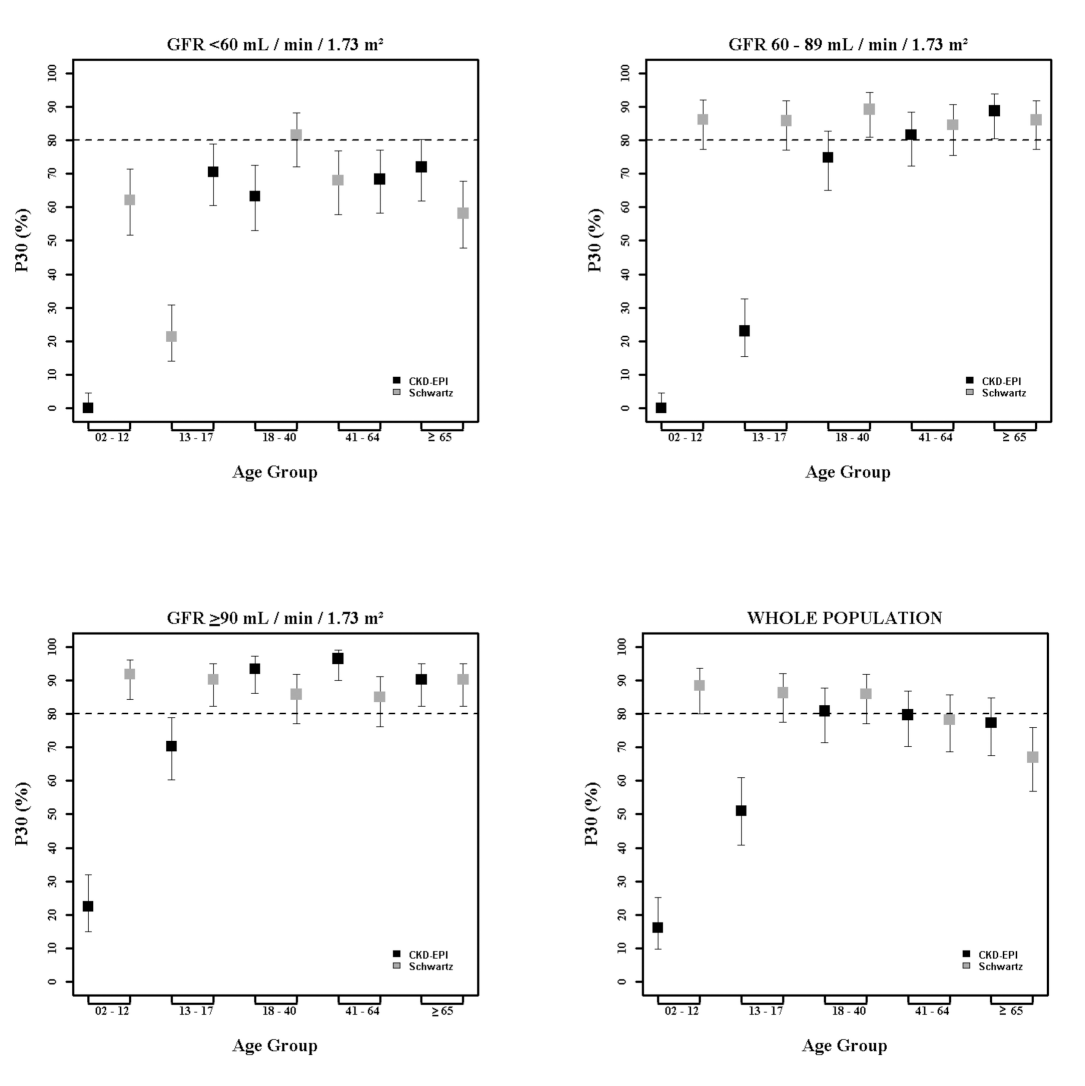
Estimation of P30 accuracy of the CKD-EPI and Schwartz equations according to age class and mGFR.

**Fig 3:**
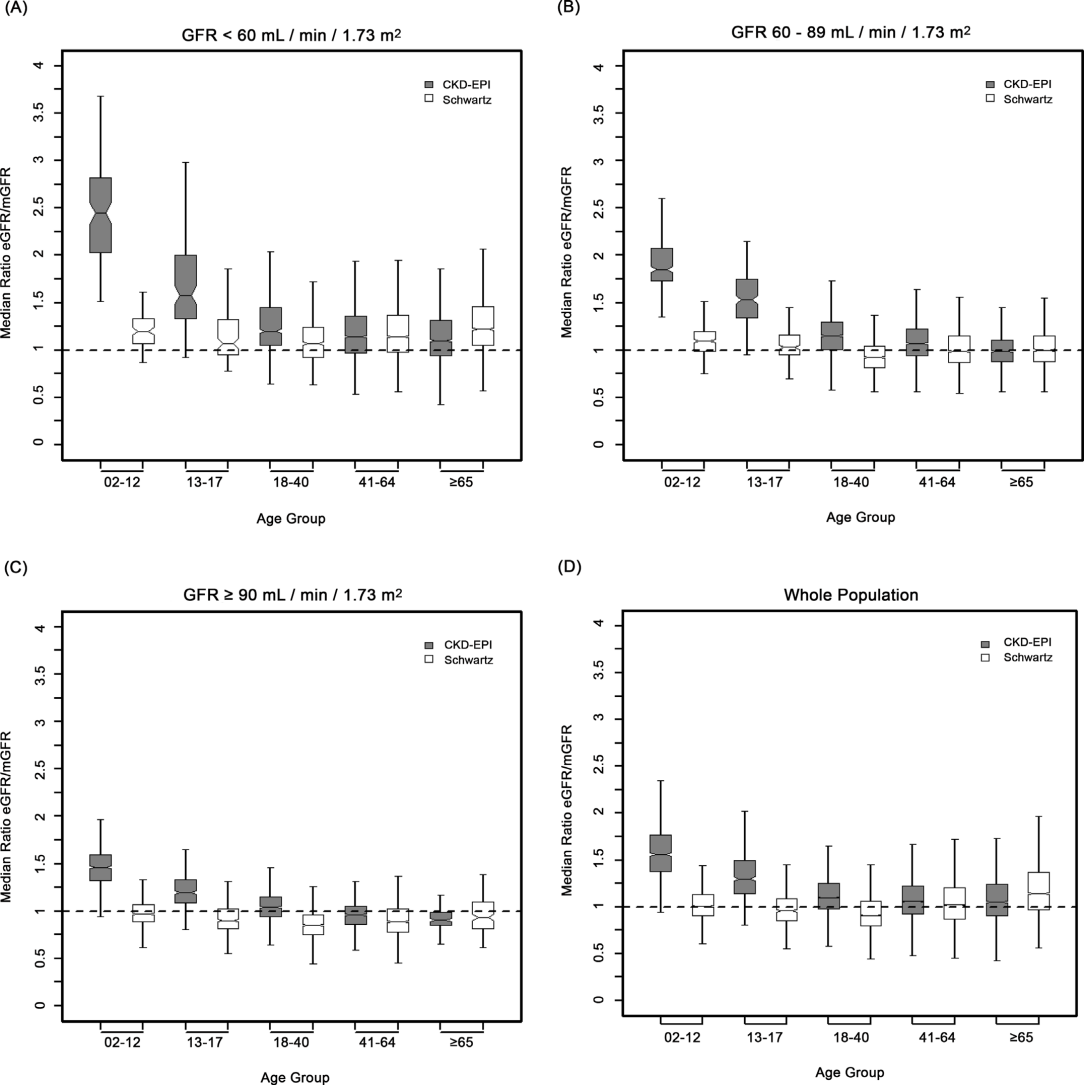
Box plots showing eGFR/mGFR ratios according to age class and stage of chronic kidney disease.

In children and adolescents (<18 y), the Schwartz equation performed better than the CKD-EPI equation regarding precision (IQR: 0.23 [95% CI 0.21; 0.24] versus 0.33 [95% CI 0.31; 0.34]) and accuracy (P10: 46.7 [95% CI 44.0; 49.6] versus 8.4 [95% CI 6.0;10.0]; P30: 86.4 [95% CI 88.6; 90.4] versus 29.4 [95% CI 26.8; 32.0]) (Tables 4 and 5; Figs 2 and 3). In addition, the Schwartz equation was slightly better than the CKD-EPI equation in young adults (18–40 y) regarding precision (IQR: 0.26 [95% CI 0.25; 0.27] versus 0.27 [95% CI 0.26; 0.28]) and P30 accuracy (86.5 [95% CI 85.0; 87.8] versus 81.0 [95% CI 79.5; 82.6]) (Tables 4 and 5; Figs 2 and 3).

**Table 4:** Precision and accuracy of the CKD-EPI and Schwartz equations according to age and mGFR in children and adolescents.

mGFR Category and Performance Index	All Participants <18 y		Children (2–12 y)		Adolescents (13–17 y)	
	CKD-EPI	Schwartz	CKD-EPI	Schwartz	CKD-EPI	Schwartz
**All mGFR categories**						
IQR (95% CI)	0.33 (0.31; 0.34)	0.23 (0.21; 0.24)	0.39 (0.37; 0.42)	0.22 (0.20; 0.23)	0.35 (0.31; 0.41)	0.21 (0.22; 0.27)
P10 (95% CI)	8.4 (6.0; 10.0)	46.7 (44.0; 49.6)	2.8 (1.6; 4.0)	49.1 (45.5; 52.6)	18.1 (14.3; 21.8)	42.7 (38.1; 47.6)
P30 (95% CI)	29.4 (26.8; 32.0)	88.6 (86.7; 90.4)	16.8 (14.1; 19.0)	89.3 (87.1; 91.5)	51.4 (46.7; 56.1)	87.2 (84.0; 90.3)
**<60 ml/min/1.73 m** ^**2**^						
IQR (95% CI)	1.04 (0.90; 1.14)	0.31 (0.29; 0.45)	0.79 (0.65; 0.99)	0.26 (0.22; 0.37)	0.67 (0.46; 0.93)	0.37 (0.24; 0.61)
P10 (95% CI)	0.8 (0.0; 2.5)	33.6 (25.1; 42.1)	0.0 (0.0; 0.0)	31.1 (19.1; 42.0)	1.6 (0.0; 5.0)	36.1 (24.0; 48.1)
P30 (95% CI)	10.9 (5.3; 16.5)	67.5 (58.7; 75.6)	0.0 (0.0; 0.0)	62.1 (49.5; 74.6)	21.3 (11.0; 35.5)	72.1 (61.0; 83.9)
**60–89 ml/min/1.73 m** ^**2**^						
IQR (95% CI)	0.42 (0.36; 0.46)	0.23 (0.20; 0.26)	0.35 (0.30; 0.40)	0.21 (0.18; 0.24)	0.41 (0.30; 0.51)	0.21 (0.17; 0.32)
P10 (95% CI)	3.0 (1.0; 5.1)	49.5 (45.5; 52.5)	0.0 (0.0; 0.0)	46.4 (38.8; 54.4)	7.1 (4.0; 10.2)	45.1 (36.6; 54.3)
P30 (95% CI)	10.3 (6.8; 13.9)	89.0 (85.5; 92.7)	0.0 (0.0; 0.0)	90.4 (85.3; 94.8)	23.9 (18.7; 29.1)	87.6 (81.5; 93.7)
**≥90 ml/min/1.73 m** ^**2**^						
IQR (95% CI)	0.32 (0.31; 0.34)	0.21 (0.19; 0.23)	0.28 (0.26; 0.31)	0.18 (0.17; 0.21)	0.24 (0.21; 0.27)	0.20 (0.17; 0.24)
P10 (95% CI)	11.2 (9.0; 13.5)	49.5 (45.5; 52.5)	3.8 (2.2; 5.5)	51.7 (47.5; 56.0)	27.0 (21.5; 32.4)	43.4 (37.3; 49.4)
P30 (95% CI)	38.5 (35.5; 41.8)	91.2 (89.6; 93.5)	23.3 (19.7; 26.8)	92.0 (89.8; 94.3)	70.7 (65.1; 72.3)	90.6 (87.0; 94.2)

IQR: IQR of the ratio of eGFR to mGFR; P10 and P30: within 10% and 30% limits above and below the mGFR.

**Table 5:** Precision and accuracy of the CKD-EPI and Schwartz equations according to age and mGFR in adults (≥18 y).

mGFR Category and Performance Index	All Adults		18–40 y		41–64 y		≥65 y	
	CKD-EPI	Schwartz	CKD-EPI	Schwartz	CKD-EPI	Schwartz	CKD-EPI	Schwartz
**All categories**								
IQR (95% CI)	0.30 (0.29; 0.31)	0.33 (0.33; 0.35)	0.27 (0.26; 0.28)	0.26 (0.25; 0.27)	0.30 (0.29; 0.31)	0.34 (0.32; 0.35)	0.33 (0.31; 0.34)	0.40 (0.38; 0.41)
P10 (95% CI)	38.1 (37.1; 39.0)	33.0 (32.3; 34.2)	38.3 (36.3; 40.2)	33.5 (31.7; 35.6)	38.5 (37.1; 39.8)	34.1 (32.8; 35.5)	36.7 (34.5; 38.9)	30.3 (28.2; 33.4)
P30 (95% CI)	79.8 (79.0; 80.6)	77.9 (77.6; 79.3)	81.0 (79.5; 82.6)	86.5 (85.0; 87.8)	80.0 (79.0; 81.1)	78.7 (77.6; 79.8)	77.6 (75.7; 79.5)	67.5 (65.4; 69.7)
**<60 ml/min/1.73 m** ^**2**^								
IQR (95% CI)	0.39 (0.38; 0.40)	0.40 (0.38; 0.41)	0.40 (0.36; 0.44)	0.32 (0.28; 0.33)	0.39 (0.38; 0.42)	0.39 (0.36; 0.41)	0.41 (0.40; 0.48)	0.41 (0.40; 0.48)
P10 (95% CI)	30.3 (28.8; 31.7)	29.4 (28.0; 31.0)	25.4 (21.7; 29.1)	35.0 (31.0; 39.1)	30.0 (26.1; 34.0)	29.8 (27.7; 31.8)	32.4 (30.0; 35.0)	26.4 (24.0; 28.8)
P30 (95% CI)	69.1 (67.8; 70.6)	67.1 (65.7; 68.7)	63.8 (59.7; 68.0)	81.4 (78.1; 84.7)	68.5 (64.5; 72.5)	68.7 (66.7; 70.7)	72.4 (70.0; 74.8)	58.7 (56.3; 61.4)
**60–89 ml/min/1.73 m** ^**2**^								
IQR (95% CI)	0.29 (0.28; 0.30)	0.26 (0.25; 0.27)	0.29 (0.27; 0.31)	0.22 (0.21; 0.24)	0.28 (0.27; 0.30)	0.28 (0.26; 0.29)	0.23 (0.22; 0.25)	0.27 (0.25; 0.28)
P10 (95% CI)	37.7 (36.3; 39.3)	38.8 (37.1; 40.5)	32.1 (28.7; 35.5)	37.7 (34.1; 41.1)	37.8 (35.7; 40.0)	39.2 (37.1; 41.4)	45.5 (41.1; 49.8)	40.0 (34.7; 43.1)
P30 (95% CI)	81.5 (80.1; 82.8)	86.3 (85.1; 87.5)	74.8 (71.6; 80.0)	89.7 (87.5; 92.0)	82.0 (80.3; 83.7)	85.0 (83.4; 86.7)	89.0 (86.3; 91.7)	86.4 (83.4; 89.4)
**≥90 ml/min/1.73 m** ^**2**^								
IQR (95% CI)	0.20 (0.20; 0.22)	0.22 (0.21; 0.24)	0.32 (0.28; 0.33)	0.40 (0.36; 0.44)	0.19 (0.18; 0.20)	0.24 (0.23; 0.26)	0.13 (0.11; 0.18)	0.27 (0.22; 0.31)
P10 (95% CI)	51.4 (49.3; 53.4)	32.3 (30.1; 34.6)	48.8 (45.7; 51.7)	30.1 (27.4; 32.8)	53.8 (51.7; 56.0)	33.4 (31.3; 35.4)	50.0 (38.5; 61.5)	37.5 (26.3; 48.6)
P30 (95% CI)	95.1 (94.2; 96.0)	86.3 (84.8; 87.7)	93.6 (92.1; 95.1)	86.6 (84.6; 88.7)	96.6 (95.8; 97.4)	85.6 (84.1; 87.2)	90.3 (83.4; 97.1)	90.3 (83.4; 97.1)

IQR: IQR of the ratio of eGFR to mGFR; P10 and P30: within 10% and 30% limits above and below the mGFR.

For individuals aged 41–64 y, the CKD-EPI equation performed better than the Schwartz equation regarding precision and P10 accuracy (IQR: 0.30 [95% CI 0.29; 0.31] versus 0.34 [95% CI 0.32; 0.35]; P10: 38.5 [95% CI 37.1; 39.8] versus 34.1 [95% CI 32.8; 35.5]; though P30 accuracies were close: 80.0 [95% CI 79.0; 81.1] versus 78.7 [95% CI 77.6; 79.8]). In patients aged 65 y and older, the CKD-EPI equation performed notably better than the Schwartz equation regarding precision and accuracy (IQR: 0.33 [95% CI 0.31; 0.34] versus 0.40 [95% CI 0.38; 0.41]; P10: 36.7 [95% CI 34.5; 38.9] versus 30.3 [95% CI 28.2; 33.4]; P30: 77.6 [95% CI 75.7; 79.5] versus 67.5 [95% CI 65.4; 69.7]) (Tables 4 and 5; Figs 2 and 3).

### Equation Performance According to mGFR Category

At mGFR < 60 ml/min/1.73 m^2^, the Schwartz equation performed better than the CKD-EPI equation in the age class 18–40 y (in patients with mGFR < 60 ml/min/1.73 m^2^, IQR: 0.32 [95% CI 0.28; 0.33] versus 0.40 [95% CI 0.36; 0.44]; P10: 35.0 [95% CI 31.0; 39.1] versus 25.4 [95% CI 21.7; 29.1]; P30: 81.4 [95% CI 78.1; 84.7] versus 63.8 [95% CI 59.7; 68.0]). In contrast, in the adult (≥18 y) population with mGFR ≥ 90 ml/min/1.73 m^2^, the CKD-EPI equation was more accurate than the Schwartz equation (IQR: 0.20 [95% CI 0.20; 0.22] versus 0.22 [95% CI 0.21; 0.24]; P10: 51.4 [95% CI 49.3; 53.4] versus 32.3 [95% CI 30.1; 34.6]; P30: 95.1 [95% CI 94.2; 96.0] versus 86.3 [95% CI 84.8; 87.7]). Finally, in adults ≥65 y of age, the CKD-EPI equation performed better than the Schwartz equation whatever the mGFR category.

## Discussion

This study compared CKD-EPI and Schwartz equation estimations of the GFR in 10,610 participants referred to a single university hospital. In this cohort, the Schwartz equation was more reliable and accurate in children and adolescents (2–17 y) and in young adults (18–40 y) with mild to moderate kidney impairment. These results may help in determining a cutoff age for switching from a pediatric to an adult equation. Up to now, the CKD-EPI equation, recommended in adults, has assumed that GFR decline starts at the age of 18 y, which may not be accurate.

GFR is currently considered the best indicator of kidney function. Direct GFR measurement being complex, PCr-based estimating equations have been proposed. KDIGO recommends using the Schwartz equation in children and the CKD-EPI equation in adults [2,4]. Developed in children and young to middle-aged adults, respectively, these equations are mathematically different and thus suffer from a lack of continuity over age, especially at the transition from childhood to adulthood. There is therefore confusion over the age at which a physician may or should switch from a pediatric to an adult eGFR equation. The present analysis compared the eGFR values given by the two equations in a large cross-sectional sample of participants who underwent clearance measurements. It investigated measurement bias across a wide range of eGFR values in successive age classes and three categories of mGFR level, which may help resolve the current confusion.

Numerous studies have demonstrated the validity of GFR-predicting equations in middle-aged adults and in children [2,4,7–11,13], but few have specifically searched for the most reliable equation in young adults (18–40 y); there is currently no consensus regarding the use of these equations in this population [11,12,14]. Additionally, a few studies have searched for a specific equation that could replace the pediatric equation in young adults [11–13].

Age-associated changes in renal function have been under study for over half a century [17,19]. GFR increases with age because of proportional kidney and body growth. However, with adjustment for body surface area, children GFR values start to reach adult values by 2 y of age, and the adjusted GFR remains constant until adulthood [17,18]. Thus, the decrease of GFR with age would begin between 30 and 40 y of age and accelerate after age 65–70 y [17–19,23]. The average decline in GFR has been estimated at 0.96 ml/min/y, or about 10 ml/min/decade [23]. Also, it has been shown that height is a good indicator of renal volume, and thus of adjusted GFR, until the sixth decade of life [24,25]. We can therefore assume that a height-dependent GFR estimating equation may be valid in young as in middle-aged adults.

Most GFR estimating equations have been developed using a combination of demographic and clinical variables [2,4]. In fact, in adult equations, such as the Modification of Diet in Renal Disease or CKD-EPI equation, age is used as a central component to integrate muscle mass decrease with age. For a given PCr value, this results in higher eGFR values in young than in old individuals [4]. The opposite is true for children; their muscle mass increases with age and is more closely correlated with height than with age [7]. In young adults, muscle mass does not begin to decrease at 18 y but remains probably constant until middle-age adulthood [2,4]. Therefore, in the CKD-EPI equation, the use of age leads to an overestimation of GFR in children, adolescents, and young adults, whereas, in people ≥65 y old, the CKD-EPI equation performs better than the Schwartz equation whatever the GFR level because of the significant muscle mass decrease.

We hypothesized that the better reliability of the Schwartz compared to the CKD-EPI equation was due to the choice of height as a surrogate for muscle mass and renal volume, and thus for GFR, which would confirm previous studies [24,25]. In these studies, “height/PCr” had a key place in building GFR estimating equations. Schwartz [15] has shown that “height/PCr” could explain more than 70% of the variability of GFR in children and young adults, probably because height is a good surrogate for muscle mass, and thus for creatinine production. In the CKD-EPI equation, age, sex, and ethnicity capture only some of the factors that affect PCr [4,8].

Despite its large scale, the present study has some limitations. First, the study population included few non-white participants and could not assess the effect of ethnicity; however, recent studies have reported that GFR is independent of ethnicity and that the use of population-specific corrections for PCr provide robust adjustments for standard eGFR equations [5,26]. Second, the performance of eGFR equations in participants with mGFR < 30 ml/min/1.73 m^2^ could not be independently examined because of the small number of participants with severe CKD.

The present analyses show that the Schwartz equation is less biased than the CKD-EPI equation in almost all subgroups. In particular, bias was low in subgroups at mild and high risk of CKD, in which an overestimation of eGFR may have led to an underestimation of CKD prevalence, including people aged less than 65 y. This has important clinical implications for eGFR reporting by laboratories and its interpretation by physicians. The better performance of the Schwartz equation should improve clinical decision-making in people with low mGFR, especially mGFR < 60 ml/min/1.73 m^2^. These people are exposed to increased risks of all-cause and cardiovascular mortality and to other traditional risk factors. However, the effect of more precise GFR estimates at lower mGFR values on clinical management should be assessed to identify and prevent the risks of CKD [2]. From another perspective, the Schwartz equation requires no additional laboratory costs and is easy to use, especially in developing countries (requires no computerized algorithms).

One interesting extension of the present report would be to conduct the same study in individuals of other ethnicities. Another wider project would be to elicit the collaboration of several centers to develop an equation able to estimate GFR across all ages and all CKD stages.

### Conclusion

In this cohort, the Schwartz equation was less biased and showed higher precision and accuracy than the CKD-EPI equation in children and adolescents whatever the level of GFR and in adults aged 18–40 y with a GFR < 90 ml/min/1.73 m^2^. These results may help determine the cutoff age at which a physician should switch from the Schwartz to the CKD-EPI equation and suggest that the Schwartz equation can be an excellent alternative that does not need a computer.

## Supporting Information

S1 Text Statistical analysis plan. (DOC)

S2 Text STARD checklist. (DOC)

## Abbreviations

BMI: body mass index
CKD: chronic kidney disease
CKD-EPI: Chronic Kidney Disease Epidemiology Collaboration
eGFR: estimated glomerular filtration rate
GFR: glomerular filtration rate
IDMS: isotope dilution mass spectrometry
IQR: interquartile range
KDIGO: Kidney Disease Improving Global Outcomes
mGFR: measured glomerular filtration rate
PCr: plasma creatinine
