# Supplementary material for: Comparison of the Schwartz and CKD-EPI Equations for Estimating Glomerular Filtration Rate in Children, Adolescents, and Adults: A Retrospective Cross-Sectional Study
Source: PLoS Med. 2016 Mar 29;13(3):e1001979. doi: 10.1371/journal.pmed.1001979 (PMC4811544; doi:10.1371/journal.pmed.1001979)
Supplement: S1 Text — (DOC) [file pmed.1001979.s001.doc]

**STATISTICAL ANALYSIS PLAN**

1. **OBJECTIVES**

The objectives of the study were the following: (1) assess the reliability of Schwartz and CKD-EPI equations in estimating the glomerular filtration rate (GFR) in a large population of subjects; and (2) assess the reliability of these equations across the whole age spectrum, including the transition from adolescence to adulthood.

1. **DATA SOURCE**

**Subjects and study design**

The study used a dataset of consecutive subjects, 3 to 90 years old, referred between July 2003 and July 2014 to a single university hospital to undergo GFR measurement for suspected or established renal dysfunction, renal risk, or before kidney donation. During the study period, some patients could have several GFR measurements but, for the present study, only the first GFR measurement in each patient was kept for analysis. The exclusion criteria were: (1) being treated with dialysis at the time of the study; (2) taking cimetidine, trimethoprim or intravenous injections of albumin or diuretics before GFR measurement.

**Modifications carried out in the revised version**

We have added two exclusion criteria: (3) GFR>160 mL/min/1.73 m^2^ (request of reviewer 2) and (4) GFR was measured by iohexol clearance (request of reviewer 3). Finally, 10,610 subjects were enrolled.

The consent form included information on the procedure itself as well as on the possibility of later use of the data for research purposes. According to the current French laws, an observational study that does not change routine management of patients does not need to be declared or submitted to the opinion of a research ethics board (Loi Huriet-Sérusclat 88-1138, 20 December 1988 and its subsequent amendments, text available at <http://www.chu-toulouse.fr/IMG/pdf/loihuriet.pdf>).

**Subgroup definition**

Reliability assessment and comparisons between the two GFR equations were carried out on pre-specified age groups and GFR categories. According to previous studies on kidney physiology, the study considered five age groups (2-12, 13-17, 18-40, 41-64, and ≥65 years). These age classes were selected as those of childhood, adolescence, early adulthood, late adulthood, and old age. Besides, various studies have shown that the GFR declines steadily with aging, starting between age 30 to 40 and accelerating after age 65 to 70 [3-6].

Because the study population included very different numbers of patients per age class in terms of KDIGO categories IIIa, IIIb, and IV, we considered only three categories of renal function (<60, 60-89, and ≥90 mL/min/1.73 m^2^). According to the KDIGO guidelines, these categories are the most prevalent in the general population [2].

1. **ESTIMATION OF THE GFR**

The eGFR was calculated according to the revised Schwartz formula and the combined CKD-EPI formula, and expressed in mL per minute per 1.73 m^2^ as follows.

| **CKD-EPI** |  |  |
| --- | --- | --- |
|  | Female; PCr ≤61.88 µmol/L | eGFR = 144 x [PCr(µmol/L)/61.88]^-0.329^ x [0.993]^Age^ x [1.159 if black] |
|  | Female; PCr >61.88µmol/L | eGFR = 144 x [PCr(µmol/L)/61.88]^-1.209^ x [0.993]^Age^ x [1.159 if black] |
|  | Male; PCr ≤79.56 µmol/L | eGFR = 141 x [PCr(µmol/L)/79.56]^-0.411^ x [0.993]^Age^ x [1.159 if black] |
|  | Male; PCr >79.56 µmol/L | eGFR = 141 x [PCr(µmol/L)/79.56]^-1.209^ x [0.993]^Age^ x [1.159 if black] |
| **Schwartz** |  | eGFR = 36.5 x height (cm) / PCr (μmol/L) |

PCr is expressed in µmol per liter and age in years.

1. **HANDLING OF MISSING VALUES AND OTHER DATA CONVENTIONS**

The data were extracted from a retrospective database. There were no missing data regarding the variables needed for the study.

1. **STATISTICAL METHODOLOGY**

**Initial statistical methods**

The reliability of each equation was assessed in terms of bias, precision, and accuracy. Classically, bias and precision are assessed using the difference between the studied method and the reference method. Plotting the difference between eGFR and mGFR according to the GFR value allowed showing an increase in inter-subjects variability of the difference with the increase of the GFR (with the two equations). The use of the eGFR/mGFR ratio rather than the difference allowed stabilizing the variance.

Bias was defined as the median ratio. Bias values below 1 indicate an underestimation of the mGFR and values above 1 an overestimation. The limits of its 95 % confidence interval (CI) were based on the normal approximation of the distribution of the estimated median.

Precision was assessed by the interquartile range (IQR) of the eGFR/mGFR ratio. The limits of its 95% CI was obtained by bootstrap using the percentiles method. This method consists of taking the 2.5^th^ and 97.5^th^ percentiles of the distribution of the IQRs calculated on 2,000 bootstrap samples.

According to the KDIGO guidelines, accuracy was defined at two levels: i) P10, the percentage of eGFR values within the 10% limits above and below the mGFR; and, ii) P30, the percentage of eGFR values within the 30% limits. The P10 and P30 percentages were estimated with their 95% confidence intervals.

Bias, precision, and accuracy were estimated in the whole sample and in each category of mGFR according to the age classes.

The comparisons of the median ratios between the two equations were made using the Wilcoxon signed-rank test. The Westenberg Mood test was used to compare the IQRs. The P10 and P30 percentages given by the two equations were compared with McNemar test that allows percentage comparisons in case of matched data.

The method of Bonferroni was used to correct for multiple comparisons. The method consisted in multiplying the p-value of each test by the number of comparisons carried out. The nominal p-value ≤0.05 was used for statistical significance.

The analyses were performed with R for Windows, version 3.1.1 (R-Cran project, <http://cran.r-project.org/>).

**Modifications carried out in the revised version**

We have changed the analysis of the bias as follows. The bias was defined as the mean of the eGFR/mGFR ratio. In a first step, the eGFR/mGFR ratio was modeled according to a linear mixed model with random intercept to quantify the effect of the equation type (CKD-EPI or Schwartz equation) on the bias. The mean ratios according to the two equations were compared by a t-test in the linear mixed model. In a second step, two models were built: a first model that included variables “equation type” and “age class” and a second model that included an interaction between variables "equation type" and "age class". The second model allows quantifying the change of the effect of the equation type according to age. An ANOVA was used to compare the two nested models and conclude regarding the statistical significance of the interaction. This analysis was carried out on the entire sample and on each category of renal function.

Estimations of precision and accuracy (with their 95% confidence intervals) were given for the two equations, in each subgroup of subjects, without comparisons by statistical tests. Actually, the amended analysis needs no correction for multiple comparisons.

**ABBREVIATIONS**

PCr plasma creatinine

mGFR Measured glomerular filtration rate

eGFR Estimated glomerular filtration rate

CKD Chronic kidney disease

^51^Cr-EDTA Chromium-51 ethylenediamine tetraacetic acid

IQR Interquartile range

CI Confidence interval

KDIGO Kidney Disease Improving Global Outcomes

CKD-EPI Chronic Kidney Disease Epidemiology Collaboration

BMI Body mass index

BSA Body surface area
