# Supplementary material for: Comparison of the Schwartz and CKD-EPI Equations for Estimating Glomerular Filtration Rate in Children, Adolescents, and Adults: A Retrospective Cross-Sectional Study
Source: PLoS Med. 2016 Mar 29;13(3):e1001979. doi: 10.1371/journal.pmed.1001979 (PMC4811544; doi:10.1371/journal.pmed.1001979)
Supplement: S2 Text — (DOC) [file pmed.1001979.s002.doc]

**Checklist of STARD items – Manuscript PMEDICINE-D-15-01238R1**

| **TITLE OR ABSTRACT** |  |
| --- | --- |
| 1- Identification as a study of diagnostic accuracy using at least one measure of accuracy (such as sensitivity, specificity, predictive values, or AUC) | The study is not exactly about the diagnostic accuracy of an "index test" but about the reliability of two equations (CKD-EPI and Schwartz) in estimating the glomerular filtration rate. The study did not require the use of sensitivity, specificity, predictive values, or AUC. The reliability of the studied equations was measured in terms of bias, precision, and accuracy. |
| **ABSTRACT** |  |
| 2- Structured summary of study design, methods, results, and conclusions (for specific guidance, see STARD for Abstracts) | The abstract is structured (Background, Methods and findings, and conclusions) according to the recommendations of PLOS Medicine. |
| **INTRODUCTION** |  |
| 3- Scientific and clinical background, including the intended use and clinical role of the index test | The scientific and clinical backgrounds are fully described, including the need and use of GFR equations. |
| 4- Study objectives and hypotheses | Given in the last paragraph of the Introduction.  The objective of the study was to assess and compare the reliability of the two most used equations to estimate GFR (in children, adolescents, young adults, adults, and elderly) and determine the equation to use according to the patient's age. |
| **METHODS** |  |
| *Study design* |  |
| 5- Whether data collection was planned before the index test and reference standard were performed (prospective study) or after (retrospective study). | The retrospective character of the study is mentioned in the first lines of section M&M. |
| *Participants* |  |
| 6- Eligibility criteria | The study considered a cross-sectional retrospective sample of 10,610 subjects 3 to 90 years old referred between July 2003 and July 2014 to a single university hospital to undergo GFR measurement for suspected or established renal dysfunction, renal risk, or before kidney donation.  The exclusion criteria were: (1) treatment by dialysis at the time of the study; (2) taking cimetidine, trimethoprim or intravenous injections of albumin or diuretics before GFR measurement; (3) GFR >160 mL/min/1.73 m^2^; (4) GFR was measured iohexol clearance. |
| 7- On what basis potentially eligible participants were identified (such as symptoms, results from previous tests, inclusion in registry) | Eligible participants were identified from the database of a single department of nephrology of a university hospital |
| 8- Where and when potentially eligible participants were identified (setting, location and dates) | The setting, location, and dates are cited in the first paragraph of M&M. |
| 9- Whether participants formed a consecutive, random or convenience series | The participants formed a consecutive series of cases |
| *Test methods* |  |
| 10a- Index test, in sufficient detail to allow replication | The GFR equations used (CKD-EPI and Schwartz) are not new tests but already well-known formulas for estimating glomerular filtration. They are presented in Table 1 and references are given for obtaining more details. |
| 10b- Reference standard, in sufficient detail to allow replication | The reference standard is the renal clearance of inulin which is still considered as the gold-standard method for GFR measurement. The method used is described in the first paragraph of the section “Laboratory assessments” of M&M. |
| - Rationale for choosing the reference standard (if alternatives exist) | The reference is the measurement of GFR (mGFR) by renal clearance of an exogenous marker that is exclusively eliminated by glomerular filtration. Inulin is one of those markers, and inulin clearance is still considered as the gold-standard method for GFR measurement. The technique is described in the first paragraph of “Laboratory assessments” of M&M. |
| 12a- Definition of and rationale for test positivity cut-offs or result categories of the index test, distinguishing pre-specified from exploratory | Not applicable |
| 12b- Definition of and rationale for test positivity cut-offs or result categories of the reference standard, distinguishing pre-specified from exploratory | The use of the categories of GFR defined by the KDIGO guidelines is pre-specified. Because of the low number of subjects in some age classes, categories III and IV were merged and three categories were finally considered (<60, 60 to 89 and >=90 mL/min/1.73 m2. This is described at the end of the first paragraph of section “Data collection” of M&M. |
| 13a- Whether clinical information and reference standard results were available to the performers/readers of the index test | The estimation of GFR with the two equations is based on the measurement of plasma creatinine which is not influenced by the knowledge of clinical information or by the results of the reference standard. |
| 13b- Whether clinical information and index test results were available to the assessors of the reference standard | Inulin clearance was carried out using a standard method by a trained staff and the influence of the knowledge of clinical information on the result is very unlikely. The results of the estimated GFR (eGFR) were not available to the assessors of the reference standard. |
| *Analysis* |  |
| 14- Methods for estimating or comparing measures of diagnostic accuracy | The reliability of the two equations in estimating the GFR was assessed by the bias. Bias was defined as the mean of the eGFR/mGFR ratio. A linear mixed model was used to quantify the effect of the equation type on the bias and to quantify the change of the effect of the equation type according to the age.  In addition the precision and the accuracy of each equation were estimated. The definition of precision and accuracy, as well as all the methods used to estimate and compare them, are presented in section “Statistical analyses” of M&M. |
| 15- How indeterminate index test or reference standard results were handled | The two equations (CKD-EPI and Schwartz) were used on 10,610 subjects included in the study. |
| 16- How missing data on the index test and reference standard were handled | There is no missing data. |
| 17- Any analyses of variability in diagnostic accuracy, distinguishing pre-specified from exploratory | Bias, precision, and accuracy are estimated for pre-specified age classes and categories of GFR. |
| 18- Intended sample size and how it was determined | The study was carried out on a retrospective sample of 10,610 subjects. All the subjects available and eligible were included. |

**Checklist of STARD items – Manuscript PMEDICINE-D-15-01238R1 (continued)**

| **RESULTS** |  |
| --- | --- |
| *Participants* |  |
| 19- Flow of participants, using a diagram | Given in Figure 1 |
| 20- Baseline demographic and clinical characteristics of participants | Given in Table 2 |
| 21a- Distribution of severity of disease in those with the target condition | Not applicable. |
| 21b- Distribution of alternative diagnoses in those without the target condition | Not applicable. |
| 22- Time interval and any clinical interventions between index test and reference standard | The estimated GFR by the two equations used plasma creatinine obtained the same day as the measurement of inulin clearance. |
| *Test results* |  |
| 23- Cross tabulation of the index test results (or their distribution) by the results of the reference standard | Not applicable. |
| 24- Estimates of diagnostic accuracy and their precision (such as 95% confidence intervals) | Estimations of the bias with their 95% confidence intervals are given in Table 3. Estimations of precision and accuracy with their 95% confidence intervals are given in Tables 4 and 5 and in Figure 2. |
| 25- Any adverse events from performing the index test or the reference standard | Not applicable. |
| **DISCUSSION** |  |
| 26- Study limitations, including sources of potential bias, statistical uncertainty, and generalisability | Given in the penultimate paragraph of the text. |
| 27- Implications for practice, including the intended use and clinical role of the index test | Given in the last paragraph of the text (Conclusion). |
| **OTHER INFORMATION** |  |
| 28- Registration number and name of registry | Not applicable. |
| 29- Where the full study protocol can be accessed | All the methods are in M&M |
| 30- Sources of funding and other support; role of funders | Given under "Role of the funding source" |
